# Notch fatigue behavior: Metallic glass versus ultra-high strength steel

**DOI:** 10.1038/srep35557

**Published:** 2016-10-18

**Authors:** X. D. Wang, R. T. Qu, S. J. Wu, Q. Q. Duan, Z. Q. Liu, Z. W. Zhu, H. F. Zhang, Z. F. Zhang

**Affiliations:** 1 Shenyang National Laboratory for Materials Science, Institute of Metal Research Chinese Academy of Sciences, 72 Wenhua Road, Shenyang, 110016, P.R. China

## Abstract

Studying the effect of notch on the fatigue behavior of structural materials is of significance for the reliability and safety designing of engineering structural components. In this work, we conducted notch fatigue experiments of two high-strength materials, i.e. a Ti_32.8_Zr_30.2_Ni_5.3_Cu_9_Be_22.7_ metallic glass (MG) and a 00Ni18Co15Mo8Ti ultra-high strength steel (CM400 UHSS), and compared their notch fatigue behavior. Experimental results showed that although both the strength and plasticity of the MG were much lower than those of the UHSS, the fatigue endurance limit of the notched MG approached to that of the notched UHSS, and the fatigue ratio of the notched MG was even higher. This interesting finding can be attributed to the unique shear banding mechanism of MG. It was found that during fatigue process abundant shear bands formed ahead of the notch root and in the vicinity of the crack in the notched MG, while limited plastic deformation was observed in the notched UHSS. The present results may improve the understanding on the fatigue mechanisms of high-strength materials and offer new strategies for structural design and engineering application of MG components with geometrical discontinuities.

As one source of stress concentration, notch plays important roles in the reliability and safety designing of engineering structural components. Hence, the study on notch fatigue property of materials is very necessary. For decades, it has been widely accepted that increasing the tensile strength usually leads to the improvement of the fatigue strength of materials^1^. However, recent experimental results broke such positive correlation and indicated that with further increasing the tensile strength, especially at a high-strength level, the fatigue strength remained invariable or even decreased^2^,^3^. Therefore, materials with very high strength may not always have an excellent fatigue performance. Then, one may ask: what about the notch fatigue property in the materials with high strength?

In the present study, we selected two high-strength materials for investigation, i.e. metallic glass (MG) and ultra-high strength steel (UHSS). MG and UHSS have been considered as promising structural materials due to their excellent mechanical properties^3^,^4^,^5^,^6^,^7^. The strength and plasticity of MG are generally inferior to those of UHSS. For instance, MGs with high strength usually exhibit nearly zero ductility under tensile loading, while the tensile plasticity of UHSS with equivalent or higher strength may achieve as high as 10%^6^,^7^. Hence the stronger UHSS has more extensive applications in fields of aircraft, vehicle, bridge, etc.^8^,^9^. However, relative to UHSS, MG has a unique notch behavior under tension^10^,^11^,^12^,^13^. The results of a series of notch tensile tests of UHSS indicated that the UHSS showed “notch weakening” and “notch brittleness” accompanying with weak dislocation slipping in front of notches^14^,^15^. In contrast with UHSS, recent experimental and simulation studies^10^,^11^,^12^ on the notch effect of MGs showed that some MGs exhibited not only “notch strengthening” but also “notch toughening”, and the strengthening effect was found increasing with decreasing the stress concentration factor (*K*_t_) of the notch. On the other hand, different from the “notch brittleness” observed in UHSS, the unique “notch toughening” behavior of MG, i.e., a notch induced improvement of tensile plasticity, was attributed to the unique shear banding mechanisms^10^,^11^. The stress concentration and the negative stress gradient induced by the notch facilitate the formation of profuse stable shear bands, leading to an enhanced plasticity of MG. Appreciable macroscopic tensile plasticity in MGs has been even achieved by introducing designed artificial defects and gradient amorphous microstructures^16^,^17^,^18^,^19^,^20^. Although great efforts have been made to explore the notch behavior of MG under monotonic loading, studies on the effect of notch on fatigue behaviors were rarely carried out. In addition, the different notch tension behavior between MG and UHSS may also make one wonder: what about the notch fatigue behavior for the two different materials? To explore the answer, we conducted the three-point bending fatigue experiments using typical MG and UHSS notched samples and compared their notch fatigue properties, with our concentrations mainly focusing on the fatigue failure mechanism of notched MG.

## Results

**Figure 1:**
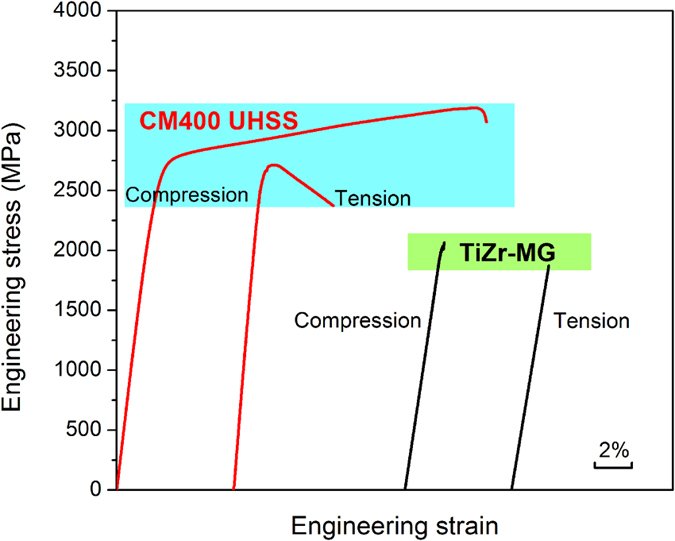
Tensile and compressive engineering stress-strain curves of the studied TiZr-based MG and CM400 UHSS.

Figure 1 shows the tensile and compressive engineering stress-strain curves for the Ti_32.8_Zr_30.2_Ni_5.3_Cu_9_Be_22.7_ (at.%) MG and 00Ni18Co15Mo8Ti (wt.%) UHSS (CM400). Obviously, the strength and plasticity for the two materials are very different. For instance, the ultimate tensile strength (UTS) of the MG, ~1873 MPa, is much lower than that of the UHSS, ~2714 MPa. And the MG exhibits nearly zero plasticity under either tension or compression, while the tensile and compressive plasticity of the UHSS are ~4% and ~20%, respectively. These results demonstrate that the UHSS has a great advantage over the MG in both the strength and plasticity.

**Figure 2:**
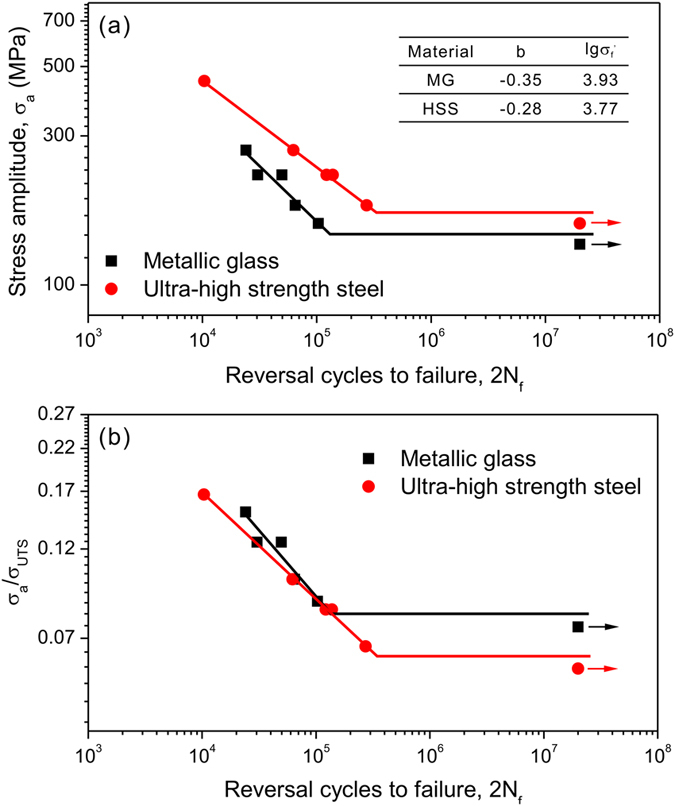
(**a**) Stress-life (S-N) fatigue data for the notched MG and the notched UHSS. Data are presented in terms of the reversal cycles to failure, 2*N*_f_, as a function of (**a**) the applied stress amplitude, *σ*_a_ = (*σ*_max_ − *σ*_min_)/2, and (**b**) the applied stress amplitude normalized by the ultimate tensile strength, i.e., *σ*_a_/*σ*_UTS_. The table in (**a**) lists the values of the fatigue coefficient, 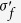, and the fatigue exponent, *b*, which were obtained by linear fitting.

The stress amplitude, *σ*_a_ = (*σ*_max_ − *σ*_min_)/2, as a function of the reversal cycles to failure, 2*N*_f_, for the notched MG and the notched UHSS were plotted in Fig. 2(a). The relationship between *σ*_a_ and 2*N*_f_ conforms the Basquin equation,


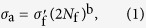


where 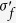 is the fatigue coefficient and *b* is the fatigue exponent. For the studied two materials, the values of 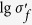 and *b* are approximately similar, as shown in the inset Table of Fig. 2(a), indicating that the fatigue life of the notched MG is close to that of the notched UHSS. Besides, the fatigue endurance limits for the notched MG and the notched UHSS, which are 145 MPa and 165 MPa, respectively, are also close. Considering the significant differences of strength and plasticity between MG and UHSS (see Fig. 1), the similar fatigue endurance limit for the two materials are quite surprising. In order to gain a more comprehensive understanding, we further plotted the relationship between the normalized stress amplitude (*σ*_a_/*σ*_UTS_) and the reversal cycles to failure (2*N*_f_), as shown in Fig. 2(b). It can be seen that above the fatigue ratio, the fatigue lives of the notched MG and the notched UHSS are nearly same. More importantly, the fatigue ratio of the notched MG (~0.08) is even higher than that of the notched UHSS (~0.06). These results demonstrate that the fatigue performance of the notched MG seems to be superior to that of the notched UHSS, although the strength and plasticity of the UHSS are much better.

## Discussion

It is worth noting that for smooth samples if only considering the fatigue ratio, the fatigue performance of typical MG may be much worse than that of typical UHSS^3^,^21^,^22^. For instance, Gilbert *et al*.^22^ reported that the fatigue ratio of a typical MG was even as low as 3–4%, while for UHSS it was reported to range from 15% to 25%^3^. This contradicts the present trend for the notched samples. Hence one may wonder why MG displays such unique notch fatigue behavior. In the following, we will elaborate the reasons based on the deformation features formed during fatigue crack initiation and propagation, the two main stages of fatigue failure.

**Figure 3:**
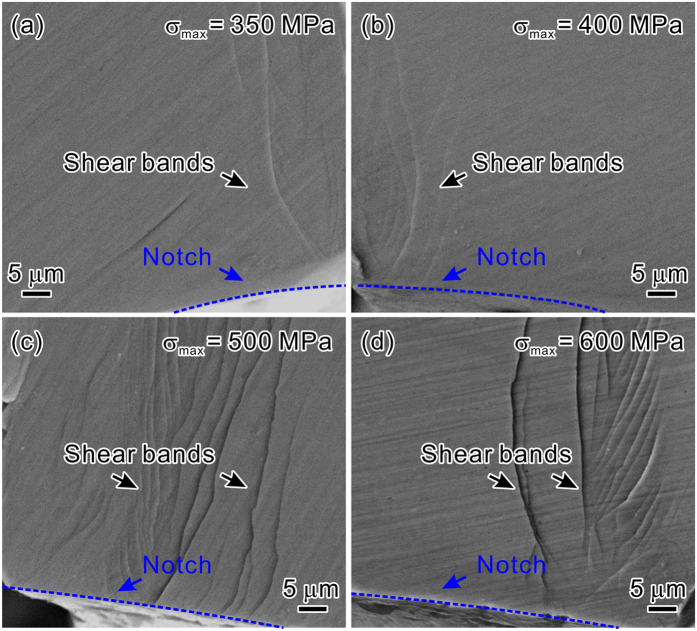
SEM observations of the deformation features at the notch roots under different nominal maximum cyclic stresses (*σ*_max_) for the notched MG. (**a**) *σ*_max_ = 350 MPa; (**b**) *σ*_max_ = 400 MPa; (**c**) *σ*_max_ = 500 MPa; (**d**) *σ*_max_ = 600 MPa. The positions of notches are indicated by the blue dashed lines.

The macroscopic deformation features in front of notch in the notched MG under different maximum cyclic stresses (*σ*_max_) are displayed in Fig. 3. Shear bands initiated from the notch root can be observed near the main crack, as indicated by the black arrows. Obviously, the density of shear bands increased with increasing the value of *σ*_max_. However, it is surprising that shear bands can form under cyclic loading with the stress level much lower than the elastic limit (*σ*_el_ = 1900 MPa) of the MG measured by monotonic compression, e.g., a *σ*_max_ of ~700 MPa calculated according to the stress concentration factor, as shown in Fig. 3(a). Our previous studies indicated that shear band could be initiated under cyclic compression at the stress level equal to or slightly lower than the elastic limit^23^,^24^. The reasons for the formation of shear bands in notched MG under cyclic stresses much lower than *σ*_el_ can be explained as follows. Recent experiments and simulations^25^,^26^,^27^,^28^ have shown that local atomic rearrangements occur in MGs under cyclic loading, which may cause the change of free volume level in forms of the exhaustion and proliferation of the fertile sites in the cyclically hardened and softened MGs. Besides, free volume content under cyclic loading is closely related to the stress state. Packard *et al*.^25^ conducted cyclic indentation experiment and suggested that the activity of free volume could be suppressed by hydrostatic pressure in MG. Nonetheless, our recent work on the cyclic softening behavior of an MG found that the cyclic compressive stress state may also trigger the free volume augmentation^29^. On the other hand, compared with the compressive stress, the tensile stress was more effective in inducing structural change by causing significantly larger atomic energy variation^30^. And molecular dynamics (MD) simulation by Cameron and Dauskardt^31^ on the fatigue damage in MG also showed that the cyclic tensile stress state would increase the overall free volume content and accelerate the formation of shear bands. Moreover, surface defects, which may enhance the stress concentration, are inevitable near the notch root. Therefore, the triaxial tensile stress state and stress concentration ahead of the notch tip under cyclic bending may promote the local proliferation of free volume more easily, and thus decrease the critical stress for shear band initiation, leading to shear banding under cyclic loading even with the stress level far below the elastic limit.

Recently, a study on the yielding of MGs by Ye *et al*.^32^ also showed that shear bands could form within the elastic stress range if a sufficient waiting time is provided. Hence the cycling frequency is expected to affect the shear banding behavior. This was proved by a recent MD simulation^33^, which showed that with decreasing the cycling frequency, shear bands were easily activated with the quick accumulation of free volume due to the adequate duration in every cycle, eventually leading to a shorter fatigue life. However, once shear bands form, the stress gradient induced by the notch and the bend loading may stabilize the shear band to progressively propagate into the sample center, resulting in profuse shear bands in front of the notch. It is worth noting that the higher applied stress level makes the local stress at the notch root easier to reach the threshold for shear band initiation; along with the action of stress gradient, more shear bands were observed, as shown in Fig. 3.

At room temperature, shear bands are not only the carriers of plastic deformation of MG but also the origins of damages and failure. It has been suggested and experimentally proved that the critical condition for shear band cracking under cyclic loading is a critical value of the local shear offset of shear band^23^,^29^. Consequently, compared with the smooth samples, the multiple shear bands at notch root in the notched MG can bear more plastic damage and then retard the initiation of fatigue crack. This increases the number of cycles needed to initiate crack, leading to an extended total fatigue life.

**Figure 4:**
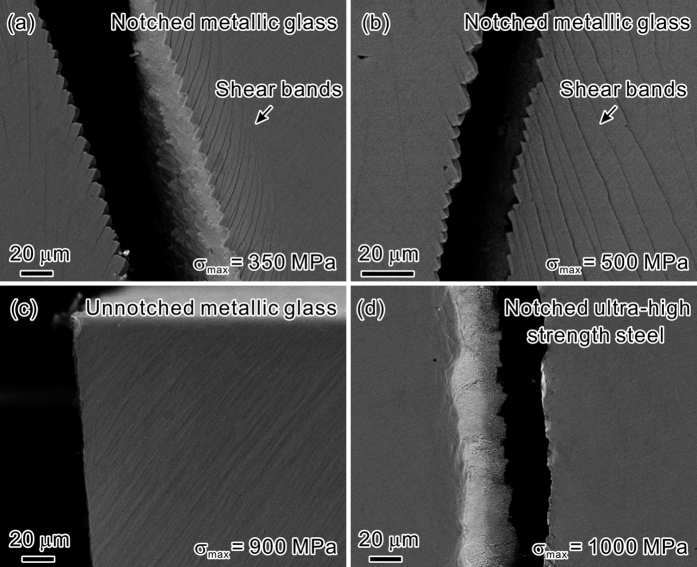
SEM observations of the fatigue crack propagation paths. “Staircase-like” crack paths with the formation of shear bands for the notched MG under (**a**) *σ*_max_ = 350 MPa and (**b**) *σ*_max_ = 500 MPa; (**c**) straight crack path without shear bands for the smooth MG fatigue sample under *σ*_max_ = 900 MPa; (**d**) straight crack path without obvious plastic deformation for the notched UHSS under *σ*_max_ = 1000 MPa.

For the fatigue crack propagation, the surface morphologies along the crack paths for the two materials are shown in Fig. 4. In the notched MG samples, deflected crack paths following a staircase-like pattern were observed under different *σ*_max_ (Fig. 4(a,b)). Abundant shear bands formed in the vicinity of the crack. These surface features are very similar to those in some high-toughness MGs^34^,^35^,^36^. For comparison, we also conducted the three-point bending fatigue experiment of the unnotched MG. As displayed in Fig. 4(c), the fatigue crack-growth trajectory of the unnotched MG is relatively flat without shear bands near the crack. This difference of crack path between notched and unnotched MG can be attributed to the effect of notch on the fatigue crack growth. When the crack emerges from the notch root and extends into the sample, it is enveloped by a fatigue transformation zone (FTZ)^37^. High concentration of free volume is generated within the zone under cyclic loading, especially at the crack tip. The notch behind the crack changes the stress distribution in front of the crack tip and enhances the level of stress concentration. As a result, more free volumes are activated, which decreases the critical stress for shear band initiation. Thus extensive shear bands form easily ahead of the crack tip at an angle larger than ~45° with respect to the tensile stress axis^38^,^39^. The stress gradient induced by the notch, crack and bending loading, suppresses the shear band propagation, and hence indirectly promotes the formation of shear bands in front of crack tip to bear the deformation. Besides, shear band is weaker than the MG matrix due to the work-softening behavior and structural damages^29^,^40^, thus acts as the path for crack propagation. Eventually, the crack may grow in a highly “zig-zag” manner. The deflected crack propagation deviates from the plane of maximum tensile stress, and the excessive shear banding at the crack tip consumes more energy. This retards the occurrence of fast fracture, and results in an increase in the fatigue crack growth life.

The above results indicate that the effects of shear banding on the fatigue crack initiation and propagation dominate the notch fatigue performance of MG. The formation and proliferation of shear bands at the notch root and the crack tip, mainly induced by the notch effect, act to lessen the notch fatigue sensitivity and enhance the fatigue resistance. Hence the fatigue crack is difficult to form and grow. In contrast, the fatigue behavior of UHSS is sensitive to external defects such as notches, holes, grooves, etc. The introduction of notch may accelerate the initiation of fatigue crack from the notch root. After initiation, the fatigue crack grows along a relatively flat path, which is nearly perpendicular to the direction of the maximum tensile stress, as shown in Fig. 4(d). In the vicinity of the crack, no obvious plastic deformation feature was observed. Therefore, the notch affects the fatigue crack initiation and propagation of the MG and the UHSS in markedly different manner, leading to their distinct fatigue performance.

**Figure 5:**
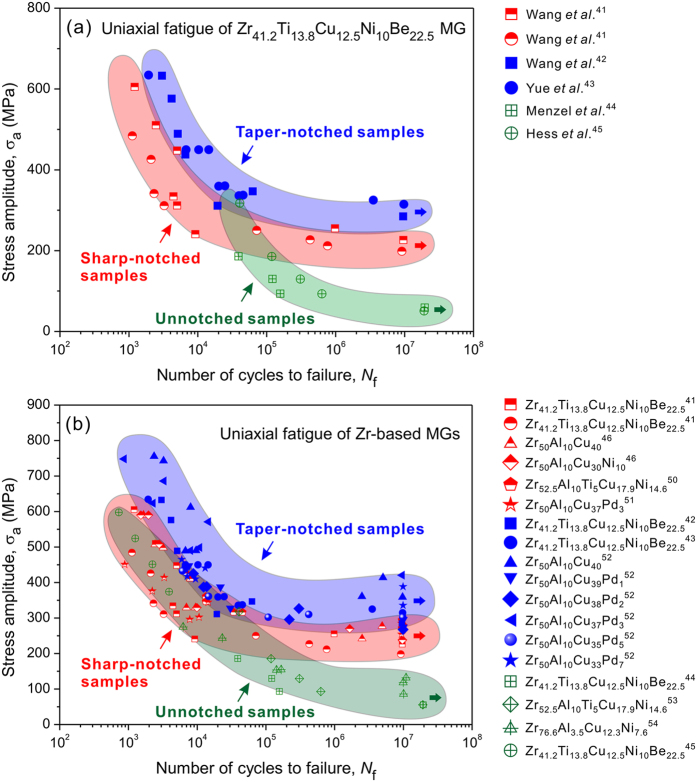
(**a**) Stress amplitude, *σ*_a_, as a function of number of cycles to failure, *N*_f_, of Zr_41.2_Ti_13.8_Cu_12.5_Ni_10_Be_22.5_ MG under uniaxial fatigue loading for different sample geometry; (**b**) *σ*_a_ as a function of *N*_f_ of various Zr-based MGs with different compositions under uniaxial fatigue loading. The data was obtained from refs 41, 42, 43, 44, 45, 46,50, 51, 52, 53, 54 and taken from the tension-tension fatigue experiments with *R* = 0.1, except for the data from Hess *et al*.^45^ who performed tension-compression fatigue experiments with *R* = −1. Note that the *σ*_a_ does not reflect the stress concentration factor at the notch root and is defined as the load amplitude divided by the reduced cross-sectional area.

To further clarify the notch effect on the fatigue property of MGs, we summarized the uniaxial fatigue data of Zr_41.2_Ti_13.8_Cu_12.5_Ni_10_Be_22.5_ MG in literature^41^,^42^,^43^,^44^,^45^ and plotted the stress amplitude (*σ*_a_) vs. the number of cycles to failure (*N*_f_) for unnotched sample and notched samples with different notch geometries, as shown in Fig. 5(a). Apparently, the fatigue strength of notched MG samples is higher than that of unnotched samples. This can be attributed to the unique notch deformation behavior in MG. SEM observations showed that few shear bands were formed in the unnotched MG, while multiple shear bands were found around the notch root under cyclic tension^46^, which is consistent with the present observations in Fig. 3. The multiple shear bands could bear more severe plastic deformation and thus retard fatigue crack initiation and propagation, leading to the longer fatigue life in the notched MG samples.

Moreover, considering the effect of notch geometry on fatigue strength, it can be found that the fatigue strength of the taper-notched MG samples^42^,^43^ (notch-root radius *r* = 24 mm and notch depth *a* = 0.51 mm) is superior to that of the sharp-notched samples^41^ (*r* = 1.27 mm and *a* = 0.51 mm), demonstrating that increasing the *r* may increase the notch fatigue strength. Actually, making the notch become blunter can also render a higher notch toughness value^47^ and a higher notch tensile strength^11^,^48^,^49^. The enhancements of the mechanical properties for the blunter notches were attributed to the more profuse and stable shear bands around notch tips^11^,^47^ and thus the larger shear band zone^12^. Analogously, under cyclic loading, the weaker stress concentration induced by the larger *r* in the taper-notched MG sample may cause a larger plastic zone with more profuse shear bands than the sharp-notched sample. Along with the action of negative stress gradient on stabilizing the progressive propagation of shear band, more inserting shear bands^24^ could form around the notch root to bear the deformation. These shear bands consume more energy and thus inhibit the fast fracture of MG under cyclic loading. Hence, the shear-banding deformation zone with a larger size in the taper-notched sample may contribute to higher fatigue strength than that of the sharp-notched sample. At last, for a more comprehensive understanding on the effect of notch on fatigue properties of MGs, the uniaxial fatigue data of both notched and unnotched samples of various Zr-based MGs with different compositions were collected^41^,^42^,^43^,^44^,^45^,^46^,^50^,^51^,^52^,^53^,^54^ and presented in Fig. 5(b). It can be readily seen that the fatigue strength of notched MGs seems to be always higher than that of unnotched MGs, and as the sharpness of notch in MGs reduces, the fatigue strength increases. The results are in good accordance with the above findings and further verify the importance of notch and notch geometry for the fatigue property of MGs.

In summary, we compared the notch fatigue behaviors of a TiZr-based MG and CM 400 UHSS. Although the strength and plasticity of the MG are much lower than those of the UHSS, the fatigue endurance limits of the notched samples are very close for the two materials, and the fatigue ratio of notched MG is even higher. The unique fatigue behavior of notched MG can be attributed to the shear banding deformation ahead of the notch root and the crack tip. Shear bands form ahead of the notch root prior to the fatigue crack initiation and its density increases as the maximum cyclic stress increases. After the initiation of the fatigue crack, it grows in a “zig-zag” manner with profuse shear bands forming in the vicinity of the crack.

These results support the shear band-mediated fatigue damage mechanisms of MGs^23^ and suggest that promoting the proliferation of shear bands and preventing the propagation of shear bands may be effective ways to enhance fatigue performance of MGs. This is consistent with the previous findings that very high fatigue endurance limit of ~0.5*σ*_UTS_ has been observed in some high-toughness MGs with extensive shear sliding at the crack tip^35^,^36^ and in MG matrix composites with abundant shear bands between second phases^55^. In addition, the present work also indicates that unlike UHSS, the unique notch fatigue behavior of MG may make the fatigue strength of MG insensitive to notches, holes and grooves, which may provide instructions for future design of MG structural components with geometrical discontinuities.

## Methods

Ti_32.8_Zr_30.2_Ni_5.3_Cu_9_Be_22.7_ (at.%) MG and 00Ni18Co15Mo8Ti (wt.%) UHSS (CM400) were selected for investigations. The amorphous structure of MG was identified by X-ray diffraction (XRD). Single edge notched rectangular beams with dimension of 7.2 mm (height) × 2 mm (thickness) × 30 mm (length) were cut by an electric spark cutting machine. All the notches were introduced along the height direction with a U-shape and notch dimensions of ~2 mm in depth and ~1 mm in notch radius. According to ref. 56, the stress concentration factor (*K*_t_) was calculated to be 2. The external surfaces of the specimens were ground and polished by 2.5 μm diamond paste. The three-point bending fatigue experiments of the notched MG and the notched UHSS samples were performed under the stress control mode by using an Instron 8871 testing machine at room temperature. A sinusoidal wave with the stress ratio *R* = *σ*_min_/*σ*_max_ = 0.1 (i.e., the ratio of the minimum cyclic stress to the maximum cyclic stress, both of which are the nominal cyclic stresses that do not reflect the stress concentration factor at the notch root) and a frequency of 40 Hz was employed for the fatigue tests. The nominal tensile stress at the notch root was calculated as,


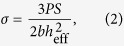


where *P* is the applied load, *b* is the sample thickness, *h*_eff_ is the effective sample height (net height between notch root and external surface), and *S* is the span. Here *h*_eff_ = 5.2 mm and *S* = 20 mm. For comparison, the three-point bending fatigue experiment of the same MG without notches was also conducted using the same sample dimensions and identical fatigue testing conditions, but only one stress level (with *σ*_max_ = 900 MPa) was tested. The surface morphologies of fatigue fractured samples were observed by a Leo Supra 35 scanning electron microscopy (SEM).

## Additional Information

**How to cite this article**: Wang, X. D. *et al*. Notch fatigue behavior: Metallic glass versus ultra-high strength steel. *Sci. Rep.* **6**, 35557; doi: 10.1038/srep35557 (2016).

## References

[b1] Tóth L. & Yarema S. Y. Formation of the science of fatigue of metals. Part 1. Mater. Sci. 42, 1825–1870 (2006).

[b2] Furuya Y. & Matsuoka S. Improvement of gigacycle fatigue properties by modified ausforming in 1600 and 2000 MPa-class low alloy steels. Metall. Mater. Trans. A 33, 3421–3431 (2002).

[b3] Pang J. C., Li S. X., Wang Z. G. & Zhang Z. F. General relation between tensile strength and fatigue strength of metallic materials. Mater. Sci. Eng. A 564, 331–341 (2013).

[b4] Inoue A. Stabilization of metallic supercooled liquid and bulk amorphous alloys. Acta Mater. 48, 279–306 (2000).

[b5] Ashby M. F. & Greer A. L. Metallic glasses as structural materials. Scripta Mater. 54, 321–326 (2006).

[b6] Zhang Z. F., Eckert J. & Schultz L. Difference in compressive and tensile fracture mechanisms of Zr59Cu20Al10Ni8Ti3 bulk metallic glass. Acta Mater. 51, 1167–1179 (2003).

[b7] Zackay V. F., Wood W. E., Goolsby R. D. & Parker E. R. Untempered ultra-high strength steels of high fracture toughness. Nat. Phy. Sci. 236, 108–109 (1972).

[b8] Lourenco J. M. . Fatigue and fracture behavior of laser clad repair of AerMet^®^ 100 ultra-high strength steel. Int. J. Fatigue 85, 18–30 (2016).

[b9] Golovashchenko S. F., Gillard A. J., Mamutov A. V., Bonnen J. F. & Tang Z. Electrohydraulic trimming of advanced and ultra high strength steels^☆^. J. Mater. Proc. Technol. 214, 1027–1043 (2014).

[b10] Qu R. T., Calin M., Eckert J. & Zhang Z. F. Metallic glasses: Notch-insensitive materials. Scripta Mater. 66, 733–736 (2012).

[b11] Qu R. T., Zhang P. & Zhang Z. F. Notch effect of materials: strengthening or weakening? J. Mater. Sci. Technol. 30, 599–608 (2014).

[b12] Sha Z. D., Pei Q. X., Liu Z. S., Zhang Y. W. & Wang T. J. Necking and notch strengthening in metallic glass with symmetric sharp- and -deep notches. Sci. Rep. 5, 10797 (2015). 26022224 10.1038/srep10797 PMC4448266

[b13] Sha Z. D. . On the notch sensitivity of CuZr metallic glasses. Appl. Phys. Lett. 103, 081903 (2013).

[b14] Hertzberg R. W. In Deformation and fracture mechanics of engineering materials. 4th edn. (ed. Santor K.) 275 (John Wiley & Sons, 1996).

[b15] Kobayashi J., Yoshikawa N. & Sugimoto K. Notch-fatigue strength of advanced TRIP-aided martensitic steels. ISIJ Int. 53, 1479–1486 (2013).

[b16] Qu R. T., Zhang Q. S. & Zhang Z. F. Achieving macroscopic tensile plasticity of monolithic bulk metallic glass by surface treatment. Scripta Mater. 68, 845–848 (2013).

[b17] Wang Q. . Superior tensile ductility in bulk metallic glass with gradient amorphous structure. Sci. Rep. 4, 4757 (2014). 24755683 10.1038/srep04757 PMC3996486

[b18] Sarac B. & Schroers J. Designing tensile ductility in metallic glasses. Nat. Commun. 4, 2158 (2013). 23863967 10.1038/ncomms3158 PMC3759052

[b19] Qu R. T., Zhao J. X., Stoica M., Eckert J. & Zhang Z. F. Macroscopic tensile plasticity of bulk metallic glass through designed artificial defects. Mater. Sci. Eng. A 534, 365–373 (2012).

[b20] Gao M., Dong J., Huan Y., Wang Y. T. & Wang W. H. Macroscopic tensile plasticity by scalarizating stress distribution in bulk metallic glass. Sci. Rep. 6, 21929 (2016). 26902264 10.1038/srep21929 PMC4763289

[b21] Menzel B. C. & Dauskardt R. H. Stress-life fatigue behavior of a Zr-based bulk metallic glass. Acta Mater. 54, 935–943 (2006).

[b22] Gilbert C. J., Lippmann J. M. & Ritchie R. O. Fatigue of a Zr-Ti-Cu-Ni-Be bulk amorphous metal: Stress/life and crack-growth behavior. Scripta Mater. 38, 537–542 (1998).

[b23] Wang X. D., Qu R. T., Liu Z. Q. & Zhang Z. F. Shear band-mediated fatigue cracking mechanism of metallic glass at high stress level. Mater. Sci. Eng. A 627, 336–339 (2015).

[b24] Qu R. T., Liu Z. Q., Wang G. & Zhang Z. F. Progressive shear band propagation in metallic glasses under compression. Acta Mater 91, 19–33 (2015).

[b25] Packard C. E., Homer E. R., Al-Aqeeli N. & Schuh C. A. Cyclic hardening of metallic glasses under Hertzian contacts: Experiments and STZ dynamics simulations. Philos. Mag. 90, 1373–1390 (2010).

[b26] Deng C. & Schuh C. A. Atomistic mechanisms of cyclic hardening in metallic glass. Appl. Phys. Lett. 100, 251909 (2012).

[b27] Packard C. E., Witmer L. M. & Schuh C. A. Hardening of a metallic glass during cyclic loading in the elastic range. Appl. Phys. Lett. 92, 171911 (2008).

[b28] Ye Y. F., Wang S., Fan J., Liu C. T. & Yang Y. Atomistic mechanism of elastic softening in metallic glass under cyclic loading revealed by molecular dynamics simulations. Intermetallics 68, 5–10 (2016).

[b29] Wang X. D., Qu R. T., Liu Z. Q. & Zhang Z. F. Shear band propagation and plastic softening of metallic glass under cyclic compression. J. Alloys Compd. Under review.

[b30] Cao R., Deng Y. & Deng C. Hardening and crystallization in monatomic metallic glass during elastic cycling. J. Mater. Res. 30, 1820–1826 (2015).

[b31] Cameron K. K. & Dauskardt R. H. Fatigue damage in bulk metallic glass I: Simulation. Scripta Mater. 54, 349–353 (2006).

[b32] Ye Y. F. . The kinetic origin of delayed yielding in metallic glasses. Appl. Phys. Lett. 108, 251901 (2016).

[b33] Sha Z. D., Qu S. X., Liu Z. S., Wang T. J. & Gao H. Cyclic Deformation in metallic glasses. Nano Lett. 15, 7010–7015 (2015). 26422317 10.1021/acs.nanolett.5b03045

[b34] Liu Y., Wang Y. M. & Liu L. Fatigue crack propagation behavior and fracture toughness in a Ni-free ZrCuFeAlAg bulk metallic glass. Acta Mater. 92, 209–219 (2015).

[b35] Song Z. Q., He Q., Ma E. & Xu J. Fatigue endurance limit and crack growth behavior of a high-toughness Zr61Ti2Cu25Al12 bulk metallic glass. Acta Mater. 99, 165–175 (2015).

[b36] Gludovatz B. . Enhanced fatigue endurance of metallic glasses through a staircase-like fracture mechanism. Proc. Natl. Acad. Sci. USA 110, 18419–18424 (2013). 24167284 10.1073/pnas.1317715110 PMC3832019

[b37] Launey M. E., Busch R. & Kruzic J. J. Effects of free volume changes and residual stresses on the fatigue and fracture behavior of a Zr-Ti-Ni-Cu-Be bulk metallic glass. Acta Mater. 56, 500–510 (2008).

[b38] Zhang Z. F. & Eckert J. Unified tensile fracture criterion. Phys. Rev. Lett. 94, 094301 (2005). 15783967 10.1103/PhysRevLett.94.094301

[b39] Qu R. T., Zhang Z. J., Zhang P., Liu Z. Q. & Zhang Z. F. Generalized energy failure criterion. Sci. Rep. 6, 23359 (2016). 26996781 10.1038/srep23359 PMC4800311

[b40] Greer A. L., Cheng Y. Q. & Ma E. Shear bands in metallic glasses. Mater. Sci. Eng. R 74, 71–132 (2013).

[b41] Wang G. Y., Landes J. D., Peker A. & Liaw P. K. Comments on “The fatigue-endurance limit of a Zr-based bulk metallic glass”. Scripta Mater. 57, 65–68 (2007).

[b42] Wang G. Y. . Comparison of fatigue behavior of a bulk metallic glass and its composite. Intermetallics 14, 1091–1097 (2006).

[b43] Yue Y. . Fatigue behavior of a Zr-based bulk metallic glass under uniaxial tension-tension and three-point bending loading mode. Intermetallics 60, 86–91 (2015).

[b44] Menzel B. C. & Dauskardt R. H. Response to comments on “The fatigue endurance limit of a Zr-based bulk metallic glass”. Scripta Mater. 57, 69–71 (2007).

[b45] Hess P. A., Menzel B. C. & Dauskardt R. H. Fatigue damage in bulk metallic glass II: Experiments. Scripta Mater. 54, 355–361 (2006).

[b46] Wang G. Y. . Fatigue behavior and fracture morphology of Zr_50_Al_10_Cu_40_ and Zr_50_Al_10_Cu_30_Ni_10_ bulk-metallic glasses. Intermetallics 12, 1219–1227 (2004).

[b47] Lowhaphandu P. & Lewandowski J. J. Fracture toughness and notched toughness of bulk amorphous alloy: Zr-Ti-Ni-Cu-Be. Scripta Mater. 38, 1811–1817 (1998).

[b48] Flores K. M. & Dauskardt R. H. Mean stress effects on flow localization and failure in a bulk metallic glass. Acta Mater. 49, 2527–2537 (2001).

[b49] Henann D. L. & Anand L. Fracture of metallic glasses at notches: Effects of notch-root radius and the ratio of the elastic shear modulus to the bulk modulus on toughness. Acta Mater. 57, 6057–6074 (2009).

[b50] Peter H. . Fatigue behavior of Zr_52.5_Al_10_Ti_5_Cu_17.9_Ni_14.6_ bulk metallic glass. Intermetallics 10, 1125–1129 (2002).

[b51] Qiao D. C. . Compression-compression fatigue and fracture behaviors of Zr_50_Al_10_Cu_37_Pd_3_ bulk-metallic glass. Mater. Trans. 48, 1828–1833 (2007).

[b52] Wang G. Y., Liaw P. K., Yokoyama Y., Freels M. & Inoue A. The influence of Pd on tension–tension fatigue behavior of Zr-based bulk-metallic glasses. Int. J. Fatigue 32, 599–604 (2010).

[b53] Zhang Z. F., Eckert J. & Schultz L. Fatigue and Fracture Behavior of Bulk Metallic Glass. Metall. Mater. Trans. 35A, 3489–3498 (2004).

[b54] Maruyama N., Nakazawa K. & Hanawa T. Fatigue properties of Zr-based bulk amorphous alloy in phosphate buffered saline solution. Mater. Trans. 43, 3118–3121 (2002).

[b55] Launey M. E., Hofmann D. C., Johnson W. L. & Ritchie R. O. Solution to the problem of the poor cyclic fatigue resistance of bulk metallic glasses. Proc. Natl. Acad. Sci. USA 106, 4986–4991 (2009). 19289820 10.1073/pnas.0900740106 PMC2663983

[b56] Pilkey W. D. & Pikey D. F. Peterson’s Stress Concentration Factors 3rd edn. (John Wiley & Sons, Hoboken, NJ, 2007).

